# Metabolic-network-driven analysis of bacterial ecological strategies

**DOI:** 10.1186/gb-2009-10-6-r61

**Published:** 2009-06-05

**Authors:** Shiri Freilich, Anat Kreimer, Elhanan Borenstein, Nir Yosef, Roded Sharan, Uri Gophna, Eytan Ruppin

**Affiliations:** 1 The Blavatnik School of Computer Sciences, Tel Aviv University, Ramat Aviv, Tel Aviv 69978, Israel; 2 School of Medicine, Tel Aviv University, Ramat Aviv, Tel Aviv 69978, Israel; 3 School of Mathematical Science, Tel Aviv University, Ramat Aviv, Tel Aviv 69978, Israel; 4 Department of Biological Sciences, Stanford University, Stanford, CA 94305-5020, USA; 5 Santa Fe Institute, Santa Fe, NM 87501, USA; 6 Department of Molecular Microbiology and Biotechnology, Faculty of Life Sciences, Tel Aviv University, Ramat Aviv, Tel Aviv 69978, Israel

## Abstract

**Background:**

The growth-rate of an organism is an important phenotypic trait, directly affecting its ability to survive in a given environment. Here we present the first large scale computational study of the association between ecological strategies and growth rate across 113 bacterial species, occupying a variety of metabolic habitats. Genomic data are used to reconstruct the species' metabolic networks and habitable metabolic environments. These reconstructions are then used to investigate the typical ecological strategies taken by organisms in terms of two basic species-specific measures: metabolic variability - the ability of a species to survive in a variety of different environments; and co-habitation score vector - the distribution of other species that co-inhabit each environment.

**Results:**

We find that growth rate is significantly correlated with metabolic variability and the level of co-habitation (that is, competition) encountered by an organism. Most bacterial organisms adopt one of two main ecological strategies: a specialized niche with little co-habitation, associated with a typically slow rate of growth; or ecological diversity with intense co-habitation, associated with a typically fast rate of growth.

**Conclusions:**

The pattern observed suggests a universal principle where metabolic flexibility is associated with a need to grow fast, possibly in the face of competition. This new ability to produce a quantitative description of the growth rate-metabolism-community relationship lays a computational foundation for the study of a variety of aspects of the communal metabolic life.

## Abstract 2

Bacterial ecological strategies revealed by metabolic network analysis show that ecological diversity correlates with metabolic flexibility, faster growth rate and intense co-habitation.

## Background

Variations in growth rate are observed both within and between species, reflecting, respectively, regulatory-level and genomic-level adaptations [1-4]. Since the rate of bacterial growth is determined by metabolic factors such as the rate and yield of ATP production [5], variations in growth rate are bound to be associated with metabolic capabilities and constraints. Several examples have demonstrated, at the single species level, that growth rate is affected by the availability of environmental resources and the level of competition in a given environment [5-8]. Comparative-growth studies have pointed to several metabolic and regulatory genes that are under selective pressure for accelerated growth - for example, genes involved in the transport of essential substrates in highly-competitive *Escherichia coli *populations [9]. However, such comparative growth studies are typically restricted to species that occupy similar ecological niches, potentially missing the impact of genomic adaptations that may vary across different niches and lifestyles. To this day, the genome design principles underlying the association between growth rate and metabolic adaptations have not yet been established at a global, cross-species scale.

A comprehensive cross-species analysis, beyond a comparative study of organisms sharing a similar ecological niche, of genomic traits that are associated with the potential growth rates of bacterial organisms was made possible due to a recent list of minimal generation times of a wide spectrum of bacterial species [10,11]. Previously, these doubling-time data have led to the important finding that variations between genes involved in translation and transcription influence growth rate [10,11]. Here we focus on the influence of genomic-derived metabolic properties. We use genomic information to generate second-order (network-based) metabolic knowledge through the reconstruction of metabolic networks, and third-order environmental knowledge through the reconstruction of habitable metabolic environments for the species studied [12]. Then, species-specific environmental information is further exploited to estimate the level of competition encountered by each organism according to the potential ability of other species to thrive in similar habitats [13]. Through converting genomic data to environmental and communal information, this study examines factors that potentially underlie growth rates across all these levels, through the analysis of the metabolic networks and environments of 528 contemporary sequenced bacterial species, where growth rate data were available for 113 of these species (Additional data file 1).

## Results and discussion

### Growth rate is associated with basic genomic and environmental attributes

We first studied the association between growth rate and the size of the genome, and the size of the corresponding metabolic network (see Materials and methods). Both attributes displayed a significant inverse correlation with doubling time (genome size, -0.31; metabolic network size, -0.38; Table 1); that is, fast growth rate is typical of species with large genomes and large metabolic networks. Notably, obligatory symbionts (parasites and mutualists) are known to have both slow growth rate and small genome size [11,14]; excluding this group from the computation, we observe no significant difference in the genome size (or network size) between slow growing and fast growing bacteria (Table 1), indicating that there is no universal link (beyond the unique properties of this group of species) between metabolic network size and bacterial growth rate. The lack of association between growth rate and genome size was already reported in previous studies [15] where the profound effects of the translation process on growth rate were suggested to mask any influences of genome size on replication speed.

**Table 1:** Correlation (*P*-value) versus duplication time

			Significance of difference between slow and fast growers^†^	
			
	Total (N = 113)	*Non-obligatory symbiont only (N = 77)	Total (N = 113)	§Non-obligatory symbionts (N = 77)
Genome size (bp)	-0.30	-0.04	0.001	0.4
	(0.001)	(0.7)	(S: 2,695,676, F: 3,402,099)	(S: 3,614,838, F: 3,479,053)
Network size	-0.38	-0.13	0.002	0.3
	(3.1e-05)	(0.2)	(S: 326, F: 410)	(S: 408, F: 431)
Fraction of	-0.42	-0.21	4e-4	0.2
regulatory genes [33]	(0.0004)	(0.13)	(S: 0.03, F: 0.05)	(S: 0.04, F: 0.05)
Estimate of	-0.34	-0.07	1e-4	-^‡^
environmental complexity [19]	(2-04)	(0.5)	(S: 3, F: 4)	
ESI	-0.25	-0.23	0.03	0.06
	(0.008)	(0.04)	(S: 0.006, F: 0.02)	(S: 0.008, F: 0.02)
ESI: random	-0.47	-0.35	8e-6	0.002
environments^§^	(1.6e-07)	(0.002)	(S: 0.007, F: 0.03)	(S: 0.01, F: 0.004)
Maximal-CHS	-0.27	-0.28	0.03	0.02
	(0.03)	(0.01)	(S: 14, F: 27)	(S: 20, F: 31)
Maximal-CHS: random	-0.34	-0.23	6e-4	0.01
environments^§^	(1e-4)	(0.05)	(S: 39, F: 72)	(S: 50, F: 85)

*According to definitions from [19] and manual curation (see Materials and methods). ^†^The two sets of data (all species and non-obligatory symbionts) were divided into two bins according to species growth rate (fast and slow). The significance between the genomic attributes studied (for example, genome size, network size, and so on) was calculated with one-sided Wilcoxon rank sum test. Values in parentheses are the mean values of the relevant attribute in the slow growing (S) and fast growing (F) groups. ^‡^Values not computed since low-ranked estimates of environmental complexity represent the obligatory symbionts, which were excluded from the analysis. ^§^Random environments are described in Materials and methods.

Moving to the environmental dimension, we examine the association between growth rate and two established measures of the variability of species' habitats (fraction of regulatory genes and environmental complexity estimate; see Materials and methods). Both measures yield similar results: a significant negative correlation with doubling time (-0.42 and -0.34, respectively; Table 1) - that is, fast growth rates are typical of species that exhibit ecological diversity. Though these correlations are insignificant following exclusion of obligatory symbionts, we still observe significant differences between fast and slow growers with respect to their fraction of regulatory genes (Table 1). While these data-driven indices track general characteristics of the environment, our goal is to focus on studying the specific relationship between metabolic factors and growth. Grouping bacterial species according to their oxygen requirements (aerobic, anaerobic, and facultative), the slowest growth rate (that is, longest mean generation time) is observed for obligatory aerobic bacteria, followed by obligatory anaerobic bacteria (Table 2). Notably, the fastest growth rate is observed for facultative bacteria (Table 2; significance over the anaerobic group, *P *= 0.03; significance over the aerobic group, *P *= 1.9e-6; Wilcoxon rank sum test); these bacteria can alternate between aerobic and anaerobic metabolism in accordance with their environment [5,8], utilizing alternative metabolic pathways to maximize rate or yield and gain an advantage over competitors [7]. The growth advantage of these facultative organisms gives rise to the hypothesis that, in general, higher growth rate may be associated with increased metabolic environmental variability and flexibility.

**Table 2:** Typical duplication time of bacterial organisms according to their mode of respiration

	Number of species*	Mean duplication time	Median duplication time	Mean network size
Aerobic bacteria	40	13	3	412
Anaerobic bacteria	18	5.3	1.6	318
Facultative bacteria	41	1.7	0.8	380

Oxygen-dependence annotations were taken from [34]. *Species in this analysis are those for which duplication times are available, the metabolic network was reconstructed (Materials and methods), and their oxygen-dependence group is one of the groups in the table (bacteria species whose oxygen-dependence annotation is 'unknown' or 'microaerophylic' are not shown here).

### Modeling metabolic-environmental attributes

**Figure 1:**
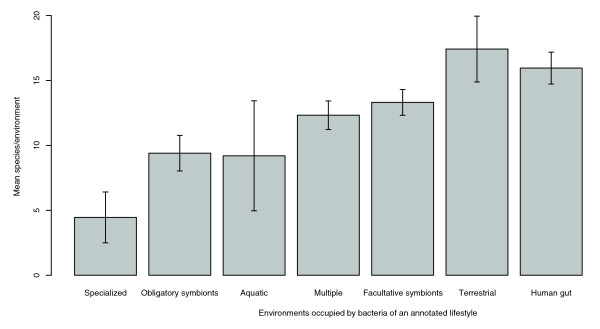
Mean co-habitation (population) levels of environments occupied by bacteria of a given lifestyle. Annotations of lifestyle are according to [19] (specialized, obligatory symbionts, aquatic, multiple, faculatative symbionts, and terrestrial) and according to identification of species in environmental samples (human gut; see Materials and methods). The number of environments in each lifestyle (in the same order as in the figure) are 11, 81, 5, 144, 157, 38, and 117 (environments can include species of more than a single lifestyle). Error bars show the standard error.

To test this hypothesis in the absence of an appropriate large-scale data-driven index of metabolic variability, we turned to develop a computational-based one. Employing a previously developed 'reverse ecology' algorithm that computes the set of metabolites that an organism extracts from its environment, we reconstructed the likely natural metabolic environments of each organism (see Materials and methods, and [12] for a comprehensive description). This provides an ensemble of environments computed for all 528 sequenced organisms, providing the broadest ecological view provided by the current data. Subsequently, the viability of each species is tested in all these environments. This is done by examining if, in a given metabolic environment (that is, a combination of metabolites), an organism can successfully expand its metabolic network so that it produces a set of target metabolites that are essential for growth (see Materials and methods). Repeating this procedure for all species provides an 'environmental viability matrix' whose rows denote the species, columns the environments, and binary entries whether a given species can survive in a given environment. We then computed the mean population level (number of species per environment) across the environments populated by organisms of a given lifestyle. Reassuringly, these results are compatible with ecological knowledge (Figure 1): soil bacteria and species populating the human gut inhabit the most densely populated environments; sparsely populated environments are inhabited by specialized organisms and (though to a lesser extent) by obligatory symbionts [16-19]. Notably, our dataset includes a large group of obligatory symbionts (54 species in comparison to 7 terrestrial organisms and 17 gut bacteria; Additional data files 1 and 2, respectively), hence indicating that the level of population of a given environment does not reflect the prevalence of the lifestyle categories of the species inhabiting it; that is, despite the ubiquity of obligatory symbionts in the data, they tend to inhabit specialized metabolic environments. We additionally examined alternative approaches for generating other biologically plausible sets of random metabolic environments. One such alternative approach for generating random environments is to construct 528 shuffled seed environments - that is, maintaining an approximation of the original metabolite representation over all seeds (see Materials and methods). Sparse populations in environments inhabited by specialized and obligatory symbionts versus dense populations in environments inhabited by soil and gut bacteria are also observed when using this alternative collection of random environments (Additional data file 3).

### Growth rate is associated with the level of metabolic variability

The 'environmental scope index' (ESI) of a species is defined as the fraction of environments in which it is viable. The ESI measure of metabolic variability is positively correlated with both genome size (0.4, *P *= 3e-6, Spearman) and metabolic network size (0.6, *P *= 1e-10, Spearman). It is also positively correlated with the data-driven general environmental-diversity measures examined above (fraction of regulatory genes, 0.32, *P *= 0.008; estimate of environmental complexity, 0.23, *P *= 0.01; Spearman), hence providing support for the ecological plausibility of the model. There is a significant negative correlation between the ESI and doubling time in the complete dataset (-0.25) and, notably, the differences between the ESI scores of slow and fast growing bacteria remain significant also after excluding obligatory symbionts (Table 1). Thus, there is a general association between broader metabolic capacities and faster maximal growth rates, extending the initial observations concerning the fast growth rate of facultative bacteria and implying that the metabolic versatility of species is better associated with their growth rate than other, more general environmental characteristics. This result, as all other reported correlations, remains valid when using the alternative collection of random environments described above (Table 1; Materials and methods). The negative correlation between ESI and duplication time is also maintained in species that are evenly distributed among different habitat types and taxonomic groups (Additional data file 3). *Pseudomonas aeruginosa*, an organism with a high ESI score (Additional data file 1) provides an example of fast growth rate in a generalist, possessing broad metabolic capabilities that allow it to successfully grow in diverse environments [20]. However, the association between fast growth and metabolic flexibility is not at all obvious, as one may assume that living in a specific niche habitat would enable an organism to specialize and adapt towards a fast growth solution. Indeed, *Desulfotalea psychrophila*, an organism with a low ESI score (Additional data file 1), provides such an example. It is a sulfate-reducing extremophilic bacterium, thriving in extreme conditions (cold arctic sediments), and exhibiting metabolic and environmental specialization [21].

### Growth rate is associated with the level of co-habitation

If metabolic specialization does not preclude fast growth, how then can we explain the slow growth of most specialists? An emerging hypothesis is that such organisms face weak competition. Conversely, organisms that occupy a large variety of metabolic environments face a larger number of co-inhabiting species, which in turn may exert selection pressure for maintaining higher growth rates. To test this hypothesis, we used the 'co-habitation score' (CHS) vector (deduced from the environmental viability matrix), denoting the number of species that co-populate each viable environment of a given species. This vector can serve as an indication of the level of competition encountered by a species in its habitats. We focus on each species' most populated niche (maximal-CHS) and most sparsely populated one (minimal-CHS). The minimal-CHS is not significantly correlated with either ESI or doubling time. In contrast, the maximal-CHS exhibits a significant inverse relationship with duplication time (Table 1) - that is, faster growth rates are observed in richly populated, competitive environments. The maximal-CHS also displays a highly marked positive correlation with metabolic variability (*P*-value < 1e-3, computed by comparing to random; Additional data file 3). That is, a species' metabolic flexibility tends to erode when it populates only sparsely populated, non-competitive environments. This result remains valid when using an alternative collection of random environments (Table 1; Materials and methods). The negative correlation between maximal-CHS and duplication time is also maintained in species that are evenly distributed among different habitat types and taxonomic groups (Additional data file 3). The relevance of maximal- and minimal-CHS to growth rate can be put in a biological context by considering the lifestyle of the pathogen *Staphylococcus aureus*: inside a host-cell (where no competition with other bacterial species is encountered) it exhibits a far slower growth rate than in the more competitive environment of the human skin [22,23].

### Delineating major ecological strategies

**Figure 2:**
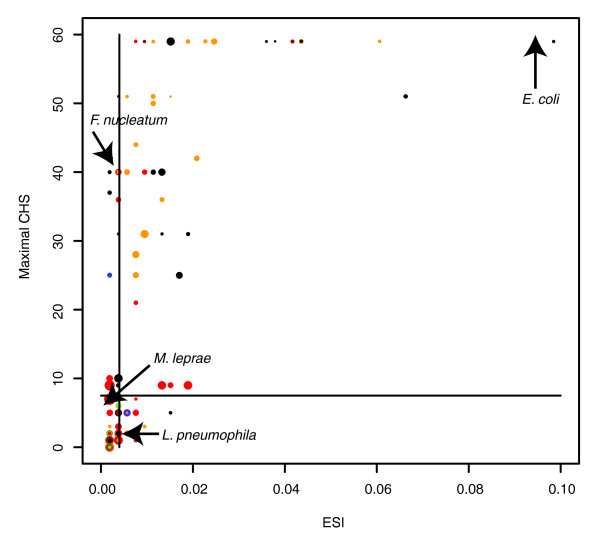
Environmental scope index versus maximal co-habitation score. The size of dots corresponds to duplication time - larger size corresponds to longer duplication time and slower growth rate. The color of dots corresponds to their ecological habitat (Additional data file 1): red, obligatory host-associated; green, specialized; blue, aquatic; black, host-associated (non-obligatory); orange, multiple; brown, terrestrial. DT, duplication time; BL, bottom left (47 species); BR, bottom right (10 species); TL, top left (16 species); TR, top right (40 species). The plot is divided according to median values of the axes.

To delineate potential major ecological strategies, we grouped the bacterial species according to their location on the ESI-CHS plane (Figure 2). As can be expected from the tight association between the environmental scope and co-habitation scores, the large majority of all species falls within the low ESI-low maximal-CHS and high ESI-high maximal-CHS diagonal groups, exhibiting two different but equally popular ecological strategies - a specialized niche with little competition or ecological diversity with intense competition, with the latter group displaying faster growth rates (*P *= 0.02, Wilcoxon rank sum test). *E. coli*, a generalist capable of fast growth, is an example of the first group, while *Mycobacterium leprae*, an obligatory intracellular pathogen with highly specialized nutritional demands and an exceptionally slow growth rate [24,25], is an example of the second group (Figure 2). However, some organisms exhibit different ecological approaches (Figure 2). In some bacterial species tight adaptation to a specific niche (low scope) does not involve escaping competition (high maximal-CHS). The oral bacterium *Fusobacterium nucleatum *is an example of a species whose metabolism is adapted to a specific, though non-exclusive, niche [26]. In contrast, the last and smallest group includes species with a relatively high environmental scope but exclusive habitats. Members of this group exhibit a faster growth rate than the low scope/low maximal-CHS group (*P *= 0.05, Wilcoxon rank sum test). As an example, the intracellular pathogen *Legionella pneumophila *has a duplication time close to a hundred times faster than *M. leprae*. Whereas *M. leprae *possesses highly specific metabolic requirements that limit its ability to exploit the resources in the host cell [25], *L. pneumophila *exhibits a more generic metabolism, scavenging the host cell for both sugars and amino acids, exhausting its own resources [27]. Accordingly, *L. pneumophila *is the causative agent of an acute disease whereas *M. leprae *causes a long-lasting chronic disease, requiring tight adaptation to co-existence within the host cell.

Beyond these specific examples, the characterization of growth strategies based on the intricate interplay between the ESI-maximal-CHS values suggests that when taken together, these values can be used for predicting growth strategies. Using ESI and maximal-CHS values retrieved from the 113 species that were included in the original analysis, we trained a support vector machine (SVM) classifier that assigns bacterial species into one of two extreme growth classes (either fast or slow; see Materials and methods). We tested the generalization ability of the classifier in a cross-validation setting, obtaining an average receiver operating characteristic (ROC) score of 0.75 (Materials and methods). We then obtained growth rate data for *Parachlamydia *UWE25 [11], an endosymbiont of amoeba from the chlamydiae group, and for *Bacillus thuringiensis*, a widely distributed bacteria [28]; for both species, growth rate data have not so far been included in our analysis. *Parachlamydia *UWE25 is an obligate intracellular bacterium that exhibits reduced central metabolic and biosynthetic pathways, and is auxotrophic for most amino acids and nucleotides [29]. In accordance with the lifestyle of this organism, we compute for this species a low environmental diversity and low competition scores (ESI, 0.002; maximal-CHS, 0; Additional data file 1). For the ubiquitous *B. thuringiensis *we compute high environmental diversity and high competition scores (ESI, 0.09; maximal-CHS, 59; Additional data file 1). We applied the SVM classifier to characterize the growth rates of these species; in accordance with experimental data, *B. thuringiensis *falls into the fast-growing category and *Parachlamydia *UWE25 falls into the slow-growing category (corresponding to the experimentally observed doubling times of 40 minutes [30] and 48 hours, respectively [11]).

## Conclusions

This paper presents the first large-scale rigorous computational exploration of the ecological strategies of a species in association with its growth rate, lifestyle and metabolic capabilities. Several limitations of this analysis should be mentioned: first, the estimation of the growth environments and their viability is based on a topological network-based computation, which is obviously a first-approximation model of the underlying biology. Second, co-habitation involves additional facets of interspecies interactions beyond competition, notably cooperation and symbiosis. Bearing this in mind, the metabolic environmental model correctly captures several patterns already observed in data-driven biological habitats, testifying to its ecological plausibility.

We find that growth rate is significantly positively correlated with both the span of the environments and the level of competition that bacterial species encounter in their metabolic habitats. The model points to two main ecological strategies, suggesting a universal principle where metabolic flexibility is associated with a need to grow fast, possibly in the face of competition. The new ability to produce a quantitative description of the growth rate-metabolism-community relationship lays a computational foundation for the study of a variety of aspects of communal metabolic life. With the growing recognition that bacteria should be better studied in the context of their ecological niche and communities, future computational approaches should take into account the complex interrelationships between organisms. Such approaches are likely to become increasingly helpful for studying various aspects of microbial life within naturally occurring ecological habitats.

## Materials and methods

### Dataset

Metabolic data were collected from the Kyoto Encyclopedia of Genes and Genomes (KEGG) [31] (release 46) for 528 bacterial organisms. Out of these species, information describing the maximal doubling time was available for 113 species (downloaded from [11]). We constructed the metabolic networks of bacterial organisms following the approach outlined in [32]. To download networks, see Additional data file 3.

### Construction of metabolic environments

Metabolic growth environments were inferred using the seed algorithm developed by [12]. This algorithm predicts the set of exogenously acquired compounds, given the metabolic network. We additionally examined alternative approaches for generating other biologically plausible sets of random metabolic environments. One such alternative approach for generating random environments is to construct 528 shuffled seed environments - that is, maintaining an approximation of the original metabolite representation over all seeds. Each metabolite in the original seed environments is randomly assigned to the shuffled environments where its representation over all environments is 1.05 times that of its original representation. That is, if a certain metabolite has, for example, 20 appearances over all seeds, then it is randomly assigned to 21 out of 528 environments. This process is repeated for each seed metabolite. The 1.05 ratio of appearances between original and shuffled environments was chosen as it allows a similar level of habitation of the environmental viability matrix, described below (Additional data file 3).

### Characterizing bacterial environments

Fraction of regulatory gene values were taken from [33], describing the fraction of transcription factors out of the total number of genes in the organism, an indicator of environmental variability [19]. Environmental complexity estimated values were obtained from [19], where the natural environments of 117 bacterial species were categorized based on the NCBI classification for bacterial lifestyle [34] and ranked according to the complexity of each category (1, obligatory symbyonts; 2, specialized; 3, aquatic; 4, facultative host-associated; 5, multiple; 6, terrestrial [19]). Annotations for environmental complexity were available for only 68 of the 113 species for which doubling time was available. To validate the reliability of these annotations, we manually searched the literature. In two cases we changed the original annotation (from multiple to terrestrial; Additional data file 4). In addition, we searched the literature for annotations for the remaining 45 species (Additional data file 4). Together with retrieving the annotations as described above, classification of species into habitats was also done by looking for the presence of species from the dataset in environmental samples. Occurrence of species from our dataset in environmental samples was inferred according to the results of a BLAST search [35] of 16S RNA sequences from the 528 species in the analysis against env_nt, a comprehensive collection of sequences from environmental samples (downloaded in February 2009). We find that in the large majority of cases (30 of 33), experimental findings support the literature-based annotations (Additional data file 3).

All parameters retrieved/computed for the species in the analysis are provided in Additional data file 1.

### Computing the environmental viability matrix, environmental scope index and co-habitation score

As a measure for species viability we constructed a list of 65 compounds termed 'target metabolites' (Additional data file 5), which are most likely essential for growth in most species [36-38]. A species-specific target metabolite list is formed by the intersection between the target metabolites and the metabolites that each species produces. We then tested the viability of each species over the set of 528 metabolic growth environments. Given a specific organism and an environment, an organism is considered viable in this environment if all its essential target metabolites are produced - this is examined by using a network expansion algorithm [39] that outputs an activated metabolic subnetwork, and verifying that the expanded subnetwork produces all target metabolites. This process yields the environmental viability matrix, whose rows denote the species, columns the metabolic environments, and binary entries the corresponding viability. From this matrix, the scope (ESI) and CHS for each species are deduced: The ESI of a species is defined as its fraction of viable environments. The CHS vector of a species records how many viable organisms populate each of its viable environments ESI and CHS values computed for all 528 species are provided (Additional data file 6).

All software used for the analysis will be provided upon request from the authors.

### Constructing a support vector machine classifier

We partitioned the organisms according to their doubling time: fast and slow growers are those whose duplication time is shorter and longer by at least one standard deviation from the mean (0.48 and 9 hours, respectively; Figure S6 in Additional data file 3). Species with intermediate values were excluded from the analysis. For the remaining species, ESI and maximal-CHS values were used for training a SVM classifier with a linear kernel [40]. We estimate the accuracy of the classifier using a ten-fold cross-validation. In this procedure, the organisms are randomly partitioned into ten distinct sets; then the class labels (slow or fast) in each set are predicted by a classifier trained on the rest of the sets. We repeated this procedure 50 times, and report the mean and standard deviation of the ROC curve [41]. Our quality metric is the area under this curve (the ROC score).

## Abbreviations

CHS: co-habitation score; ESI: environmental scope index; ROC: receiver operating characteristic; SVM: support vector machine.

## Authors' contributions

SF designed and performed research, analyzed the data and drafted the manuscript. AK performed research, analyzed the data and drafted the manuscript. EB contributed analysis tools. NY performed research. UG analyzed the data. RS analyzed the data. ER conceived and designed research and wrote the paper. All authors discussed the results and commented on the manuscript.

## Additional data files

The following additional data are available with the online version of this paper: a table listing genomic and ecological attributes for the 113 species in the analysis (Additional data file 1); a table listing the NCBI annotations and description of environmental samples for species that can be identified in an environmental sample (Additional data file 2); supplementary notes and figures, detailed description of all tables in the Additional data files and a table (Table S5) (Additional data file 3); a table listing the original and manually curated values of environmental complexity (Additional data file 4); a table listing the biomass target metabolites (Additional data file 5); a table listing genomic and ecological attributes for the 528 species in the metabolic analysis (Additional data file 6).

## Supplementary Material

Additional data file 1 Genomic and ecological attributes for the 113 species in the analysis. Click here for file

Additional data file 2 NCBI annotations and description of environmental samples for species that can be identified in an environmental sample. Click here for file

Additional data file 3 Supplementary notes and figures, detailed description of all tables in the Additional data files and a table. Table S5: Correlation (*P *value) versus duplication time in random environments. Figure S1: Distribution of the correlations between doubling time and ESI and maximal-CHS in random samples of species selected in a way that allows equal number of representatives for each ecological habitat. Figure S2: Distribution of the correlations between doubling time and ESI and maximal-CHS in random samples of species selected in a way that allows a single representative for each taxonomic group. Figure S3: The distribution of Pearson correlation coefficients of the ESI values with randomized maximal CHS values. Figure S4: General distribution of species/environment in 3 different sets of environments (original and random). Figure S5: Mean maximal CHS levels of bacteria of a given life style. Figure S6: The distribution of log doubling time of the 113 species studied. Figure S7: The mean and standard deviation of the recoever operating characteristics (ROC) curve obtained in 50 cross validation experiments. Click here for file

Additional data file 4 Original and manually curated values of environmental complexity. Click here for file

Additional data file 5 Biomass target metabolites. Click here for file

Additional data file 6 Genomic and ecological attributes for the 528 species in the metabolic analysis. Click here for file
