# Supplementary material for: Metabolic-network-driven analysis of bacterial ecological strategies
Source: Genome Biol. 2009 Jun 5;10(6):R61. doi: 10.1186/gb-2009-10-6-r61 (PMC2718495; doi:10.1186/gb-2009-10-6-r61)
Supplement: Additional data file 3 — Supplementary notes and figures, detailed description of all tables in the Additional data files and a table. Table S5: Correlation (P value) versus duplication time in random environments. Figure S1: Distribution of the correlations between doubling time and ESI and maximal-CHS in random samples of species selected in a way that allows equal number of representatives for each ecological habitat. Figure S2: Distribution of the correlations between doubling time and ESI and maximal-CHS in random samples of species selected in a way that allows a single representative for each taxonomic group. Figure S3: The distribution of Pearson correlation coefficients of the ESI values with randomized maximal CHS values. Figure S4: General distribution of species/environment in 3 different sets of environments (original and random). Figure S5: Mean maximal CHS levels of bacteria of a given life style. Figure S6: The distribution of log doubling time of the 113 species studied. Figure S7: The mean and standard deviation of the recoever operating characteristics (ROC) curve obtained in 50 cross validation experiments. [file gb-2009-10-6-r61-S3.pdf]

Additional data file

Metabolic-network driven analysis of bacterial ecological strategies

Shiri Freilich, Anat Kreimer, Elhanan Borenstein, Nir Yosef, Roded Sharan, Uri Gophna & Eytan Ruppin

Metabolic Networks

Metabolic networks are available from [42].

Supplementary Note 1

To show that the correlation observed between doubling time and ESI/maximal-CHS is not affected by an uneven representation of the diversity of habitats in nature in our dataset, we used NCBI classification to divide the species into 5 groups (Aquatic, Multiple, Terrestrial, Host-associated and Specialized). The smallest group (Specialized) is consists of 30 species. We randomly chose 30 species out of the remaining four groups (while preferring species that have a doubling time record). Using the selected 150 representatives, we built an *environmental viability matrix* and used it to calculate *ESI* and *maximal-CHS* values. In each set of 150 species doubling time information was available for 77 representatives. When computing the Spearman correlations between doubling time and *ESI* and *maximal-CHS* and in 1000 random runs we observe a negative correlation across 998 and 999 samples respectively (Figure S1). These findings imply that our results are not affected by the uneven representation of environments in our data set.

Supplementary Note 2

To show that the correlation observed between doubling time and ESI/maximal-CHS is not affected by an uneven representation of taxonomic groups in our dataset, we used KEGG taxonomic annotations to divide the species into 25 groups according to their classes (Table S1). One representative (preferably a species that has a doubling time record) was picked randomly out of each group. Using this ensemble of 25 representatives, we built an *environmental viability matrix* and used it to calculate *ESI* and *CHS* values. In each set of 25 species doubling time information was available for 22 representatives. When computing the Spearman correlations between *ESI* and doubling time, and between *CHS* and doubling for 1000 random runs, we observe a negative correlation in 934 and 969 respectively (Figure S2). These findings imply that our results are not affected by the uneven representation of taxonmic groups in our data set.

Supplementary Note 3

The ESI and max CHS measures are tightly related by definition, and expectedly are highly correlated (r = 0.579, p<1.3e-48, Pearson). To test whether this correlation is not simply due to the definition of the variables, we compute the correlation between ESI and randomized max CHS values, obtained by separately shuffling each lines of the environmental viability matrix (thus maintaining the same number of viable environments per-organism). We compute p-values in two ways: (i) using a Z-score $\frac{r-h}{\sigma_h}$ where r is the correlation index between ESI and the true max CHS, *h* is

the mean correlation in the randomized cases and σ_h is their standard deviation. (ii) Empirical p-value, calculated as the fraction of random cases in which the correlation was higher than the one observed in the original instance. In both cases the p-values are lower than 1e-3. (Figure S3).

Supplementary Note 4

The seed set of a species is the union of metabolites that a species might extract from the external world in different habitats, as discussed in detail in [12]. Based on a topological analysis of the species' metabolic network, it provides a first approximation of the species metabolic environment, and computed for each species, it provides an approximation of the ensemble of metabolic environments that the species studied here may face. Yet, we additionally examined alternative approaches for generating other biologically-plausible sets of metabolic environments (and then recalculating the corresponding *environmental viability matrix*), and studied the robustness of our main findings under these conditions.

One such alternative approach for studying the effect of the environmental composition on our observations is to create random sets of environments. The first set of random environments, Random Env I, is composed of 528 shuffled seeds environments, i.e., maintaining the original metabolites representation overall seeds. That is, if a certain metabolite has X appearances over all seeds, then it is randomly assigned to X out of 528 environments. This process is repeated for each seed metabolite. The resulting environments range in size from 259 to 329 metabolites (mean number of metabolites per environment: 295). The distribution of species per environment in the original seed data and in the randomized-source set can be seen in

Figure S4a and S4b. The mean and maximal co-habitation of environments from the random set is smaller than that of the seed environments (mean 1 and 5.7; max: 49 and 60 respectively), as expected for environments which were randomly constructed. Both ESI values and *maximal-CHS* values are in significant negative association with doubling time, repeating and reinforcing the trends reported in the main text (Table S5).

Although fast-growing bacteria exhibit higher ESI and maximal-CHS in comparison to slow growing bacteria (Table S5) – as observed while using the original seeds – the negative correlation between maximal-CHS and doubling time is insignificant following excluding the group of obligatory host-associated bacteria. One possible explanation to the lack of significance is that the environmental viability matrix is very sparse while using the shuffled environment (Figure S4). Hence we created a more densely populated set of shuffled environments, Random Env II, by increasing the number of metabolites per environment while maintaining an approximation of the original metabolites representation overall seeds. Each metabolite in the original seed environments is randomly assigned to the shuffled environments where its representation over all environments is 1.05 times in comparison to its original representation. That is, if a certain metabolite has, for example, 20 appearances over all seeds, then it is randomly assigned to 21 out of 528 environments. The distribution of species per environment in Random Env II can be seen in Figure S4c. The mean and maximal co-habitation of environments this set is higher than that of Random Env I (mean 9.7; max: 123). As in the original seed environments, the mean maximal co-habitation of gut and soil bacteria is higher than the mean maximal co-habitation of specialized and obligatory symbiont bacteria (Figure S5). In this set – creating a less sparse environmental viability matrix – we

repeat all main observation reported as well as negative correlation between doubling time and maximal-CHS following excluding the group of obligatory host-associated bacteria (Table S5).

Overall, it is reassuring to see that using several approaches for constructing potential sets of natural metabolic environments we find associations that are qualitatively similar to the ones reported in the main text.

Supplementary Note 5

We compared the annotations retrieved from NCBI [34] to the environmental sample where we identify the species. In 14 cases the sample matches the NCBI annotation (e.g., sequences annotated as aquatic or host-associated are found in marine and gut samples, respectively); in 16 cases the sample does not contradict the NCBI annotation (e.g., sequences annotated as multiple are found in marine samples); in 3 cases the experimental finding contradict NCBI annotations (in all 3 cases sequences annotated as terrestrial are found in marine samples). The 33 cases are presented at Table S2. A larger collection of environmental databases will allow a more comprehensive analysis.

Supplementary Tables

Table S1

The table displays the following values for the 113 bacterial species: name, genome size (bp), network sizes (number of reaction-nodes), *environmental scope index* (ESI), maximal *co-habitation score* (max-CHS) computed for the original seed enviroenmnets and for random environments I and II, , fraction of regulatory genes, estimates of environmental complexity, and lifestyle description (Methods), doubling time, ecological group as presented in Figure 2b (BR,BL,TR,TL), and oxygen requirements. Downloaded values: maximal doubling time[11]; genome size [11]; fraction of regulatory genes [33] and estimates of environmental complexity [19]; and description of habitats [34]. Notably, the environmental complexity class was added manually for 45 species and the original annotation was modified for 2 species, as described at Table S3. Computed values: size of the metabolic network; *ESI*; maximal-*CHS*. The *ESI* and max-*CHS* values are computed using the original seeds (Methods).

[Enclosed as Additional data file 1]

Table S2

NCBI annotations and description of environmental sample for 33 species that can be identified in an environmental sample.

[Enclosed as Additional data file 2]

Table S3

Original values of environmental complexity, as downloaded from [19], and values added by manual curation. For these values added by manual curation, reference is provided (pubmed id).

[Enclosed as Additional data file 4]

Table S4

Full description and KEGG ID of the 65 biomass target metabolites.

[Enclosed as Additional data file 5]

Table S5

Correlation (and *P* value) of duplication time vs. *ESI* and max-*CHS* values, calculated for the Random Env I and Random Env II sets of environments (Supplementary Note 4).

Table S6

The table displays the following values for the 528 bacterial species: name, genome size (bp), network sizes (number of reaction-nodes), *environmental scope index* (ESI) and maximal *co-habitation score* (max-CHS) computed for the original seed enviroenmnets, fraction of regulatory genes, estimates of environmental complexity, lifestyle description (Methods), and oxygen requirements. Values were retrieved as described for Table S1.

[Enclosed as Additional data file 6]

Table S5

Table 1. **Correlation (P value) V. Duplication time**

	Total (N=113)	§ **Non obligatory symbionts spc. (N=77)**	**Significance of difference between slow grower, fast grower±**	
			**Total (N=113)**	§ **Non obligatory symbionts spc. (N=77)**
ESI, random env I	-0.32 (5e-04)	-0.24 (0.04)	0.004 (S: 5e-004 F: 0.003)	0.02 (S: 8e-004 F:0.00 4)
Max *CHS*, random env I	-0.28 (0.002)	-0.18 0.1	0.01 (S: 5 F: 11)	0.05 (S: 8 F: 14)
ESI, random env II	-0.47 (1,6e-07)	-0.35 (0.002)	8e-6 (S: 0.007 F: 0.03)	0.002 (S: 0.01 F0.00 4)
Max *CHS*, random env II	-0.34 (1e-4)	-0.23 0.05	6e-4 (S: 39 F: 72)	0.01 (S: 50 F: 85)

±The two sets of data (all species, non obligatory symbionts) were divided into two bins according to species' growth rate (fast and slow). The significance between the genomic attributes studied (e.g., genome size, network size etc) was calculated with one-sided Wilcoxon rank sum test. In brackets: the mean value of the relevant attribute in the slow growing and fast growing groups.

§ According to definitions from [19]. Definitions were available for 68 species from the dataset; annotations for the remaining 45 species were obtained by manual curation (Table S1).

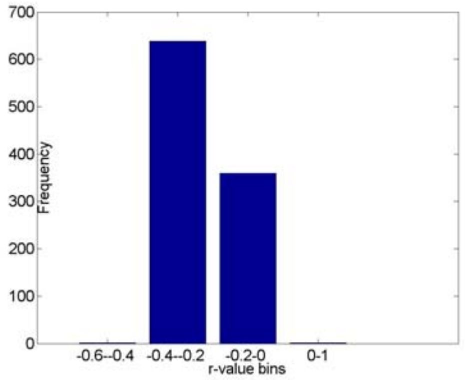

b

Figure S1. Distribution of the Spearman correlation between doubling time and ESI (A) and maximal-CHS (B) in 1000 samples of 150 species selected in a way that allows equal number of representatives for each ecological habitat (Aquatic, Multiple, Terrestrial, Host-associated and Specialized; [34])

a

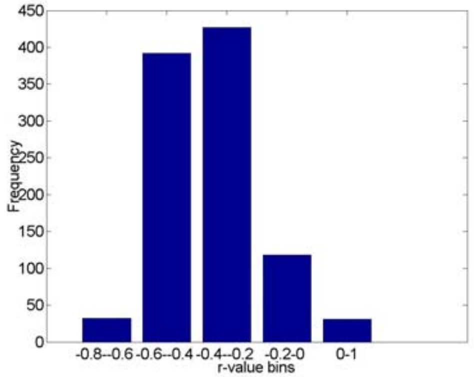

b

Figure S2. Distribution of the Spearman correlation between doubling time and ESI (A) and maximal-CHS (B) in 1000 samples of 25 species selected in a way that allows a single representatives for each taxonomic group (Family in Table S1).

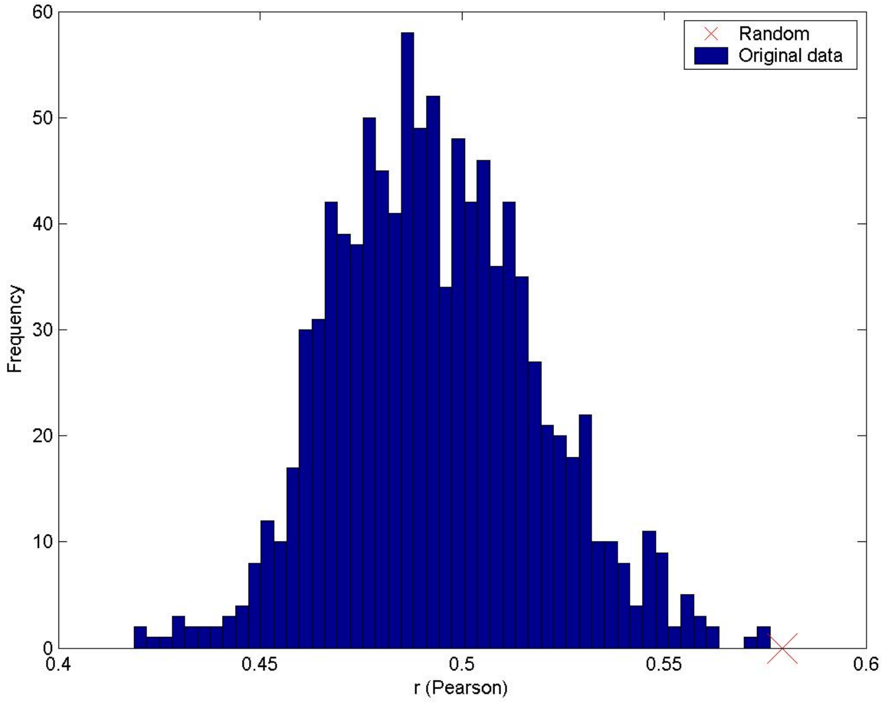

Figure S3. The distribution of Pearson correlation coefficients of the ESI values with randomized max CHS values. The red X mark denotes the Pearson correlation between the ESI score and the original max CHS score.

a

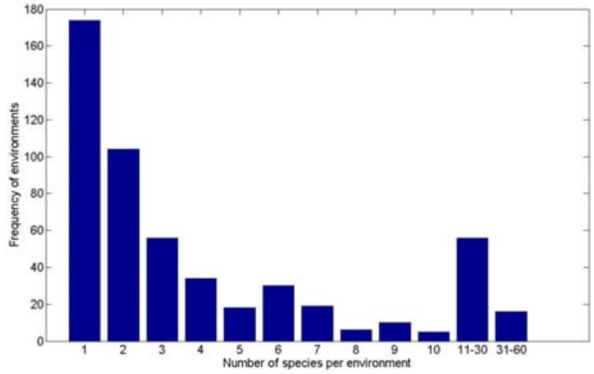

b

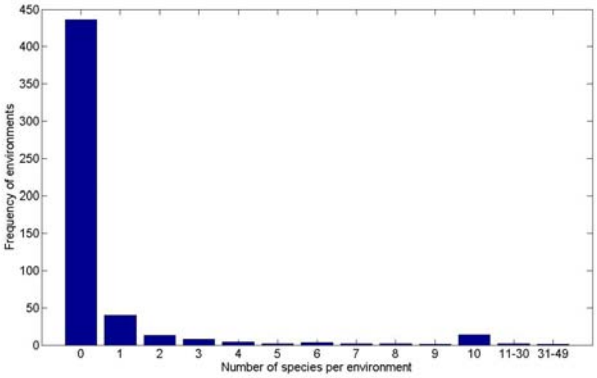

c

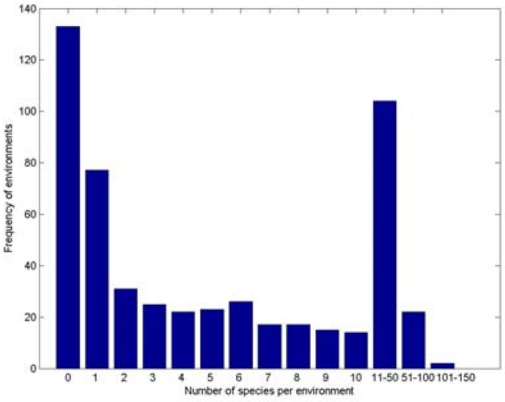

Figure S4. General distribution of species/environment of the 3 different sets of environments. (A) Original seeds environment. (B) Random Env I. (C) Random Env II.

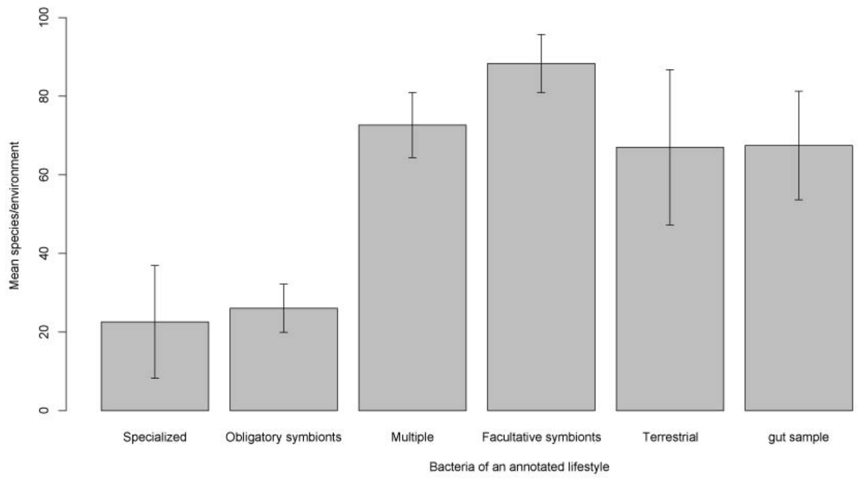

F**igure S5.** Mean maximal co-habitation levels of bacteria of a given life style. Annotations of lifestyle are as described in the main text (Figure 1). Bars show the standard error.

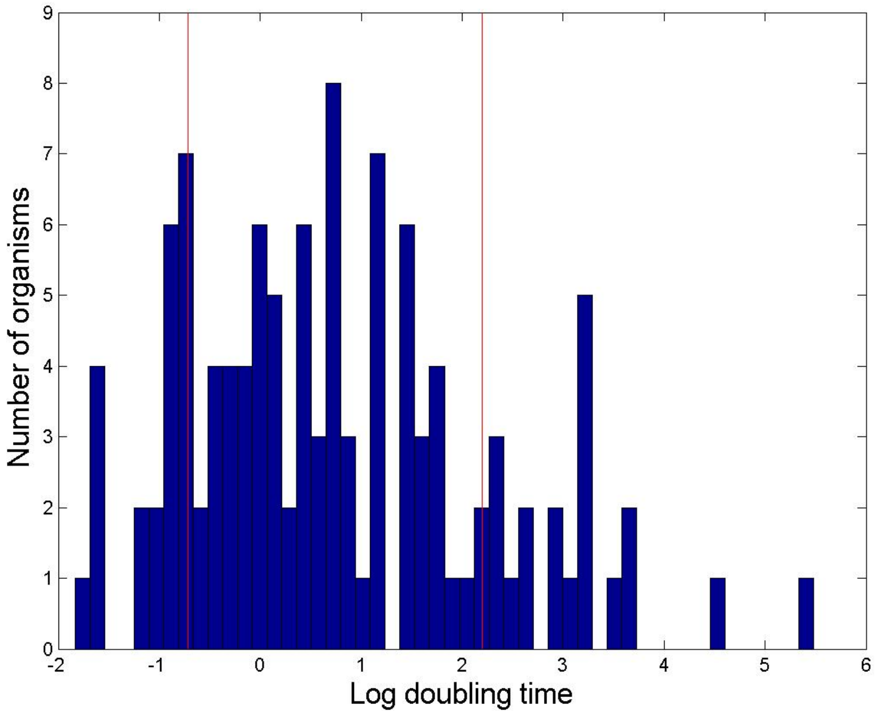

Figure S6. The distribution of log doubling time of the 113 species used. The red bars show the cutoffs for slow and fast growers.

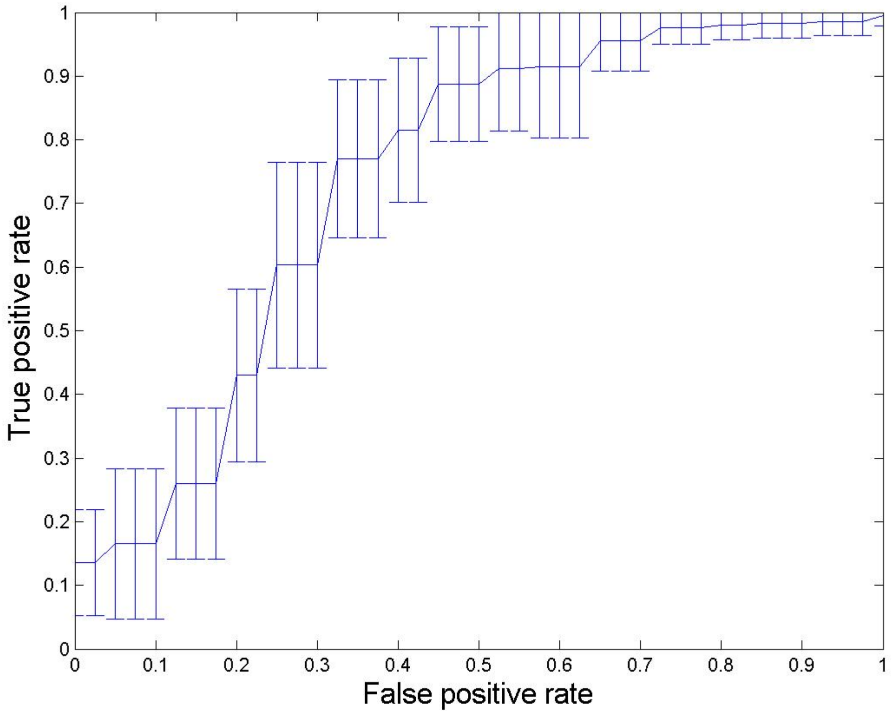

Figure S7. The mean (line) and standard deviation (bars) of the recoever operating characteristics (ROC) curve obtained in the 50 cross validation experiments.
