# Supplementary material for: Metabolic-network-driven analysis of bacterial ecological strategies
Source: Genome Biol. 2009 Jun 5;10(6):R61. doi: 10.1186/gb-2009-10-6-r61 (PMC2718495; doi:10.1186/gb-2009-10-6-r61)
Supplement: Additional data file 5 — Biomass target metabolites. [file gb-2009-10-6-r61-S5.pdf]

KEGG #	Description
C00002	ATP
C00003	NAD+
C00004	NADH
C00005	NADPH
C00006	NADP+
C00008	ADP
C00015	UDP
C00016	FAD
C00020	AMP
C00024	Acetyl-CoA
C00025	L-Glutamate
C00035	GDP
C00037	Glycine
C00041	L-Alanine
C00043	UDP-N-acetyl-D-glucosamine
C00044	GTP
C00047	L-Lysine
C00049	L-Aspartate
C00054	Adenosine 3',5'-bisphosphate
C00055	CMP
C00062	L-Arginine
C00063	CTP
C00064	L-Glutamine
C00065	L-Serine
C00073	L-Methionine
C00075	UTP
C00078	L-Tryptophan
C00079	L-Phenylalanine
C00082	L-Tyrosine
C00097	L-Cysteine
C00105	UMP
C00112	CDP
C00116	Glycerol
C00123	L-Leucine
C00131	dATP
C00135	L-Histidine
C00144	GMP
C00148	L-Proline
C00152	L-Asparagine
C00183	L-Valine
C00188	L-Threonine
C00234	10-Formyltetrahydrofolate
C00239	dCMP
C00249	Hexadecanoic acid
C00255	Riboflavin
C00286	dGTP
C00350	Phosphatidylethanolamine
C00360	dAMP
C00362	dGMP
C00364	dTMP
C00399	Ubiquinone

C00407	L-Isoleucine
C00458	dCTP
C00459	dTTP
C00641	1,2-Diacyl-sn-glycerol
C00748	Siroheme
C01050	UDP-N-acetylmuramate
C05764	Hexadecanoyl-[acp]
C05890	Undecaprenyl-diphospho-N-acetylmuramoyl-(N-acetylglucosamine)-L-
C05894	Undecaprenyl-diphospho-N-acetylmuramoyl-(N-acetylglucosamine)-L-
C05899	Undecaprenyl-diphospho-N-acetylmuramoyl-(N-acetylglucosamine)-L-
C05980	Cardiolipin
C06040	Diglucosyl-diacylglycerol
C15672	Heme O
C16221	(2E)-Octadecenoyl-[acp]
